# Posterior Reversible Encephalopathy Syndrome (PRES) in an Elderly Patient With Advanced Parkinson's Disease and Autonomic Dysfunction: A Case Report and Literature Review

**DOI:** 10.7759/cureus.104879

**Published:** 2026-03-08

**Authors:** Moustafa U Abdelradi, Awad Al Harbi, Eyas Alsuhaibani, Tibyan Amir, Aya Yasser

**Affiliations:** 1 General Practice, Dr. Sulaiman Al Habib Medical Group, Riyadh, SAU; 2 Neurosciences, King Abdullah bin Abdulaziz University Hospital, Princess Nourah Bint Abdulrahman University, Riyadh, SAU; 3 Radiology, King Abdullah bin Abdulaziz University Hospital, Princess Nourah Bint Abdulrahman University, Riyadh, SAU

**Keywords:** autonomic dysfunction, diurnal variation, parkinson's disease, posterior reversible encephalopathy syndrome, vasogenic edema

## Abstract

Posterior reversible encephalopathy syndrome (PRES) is a clinico-radiological syndrome characterized by neurological disorder and distinctive neuroimaging findings of vasogenic edema, most commonly involving the parieto-occipital regions. Blood pressure fluctuation is a common complaint in patients with Parkinson’s disease (PD), which might be explained by autonomic nervous system dysfunction, which impairs the body's ability to automatically regulate blood pressure. Additionally, PD medications can sometimes contribute to these fluctuations, leading to episodes of both low and, less commonly, high blood pressure. We report a case of an 82-year-old female patient with a longstanding history of PD who presented with symptoms of decreased level of consciousness and was found to have typical magnetic resonance imaging (MRI) characteristics of PRES. The patient had a history of blood pressure fluctuations with diurnal variation, which is a recognized autonomic dysfunction in PD. This case underscores the possible association between autonomic dysfunction in PD and the development of PRES. With appropriate blood pressure control, the patient showed both clinical and radiological improvement. This report also reviews the literature on PRES in elderly patients, autonomic dysfunction in PD, and the management considerations in such complex cases.

## Introduction

Parkinson's disease (PD) is a progressive neurodegenerative disorder primarily characterized by motor symptoms such as tremor, rigidity, bradykinesia, and postural instability [1]. However, non-motor symptoms, including autonomic dysfunction, are increasingly recognized as significant components of the disease [2]. Among these autonomic dysfunctions, blood pressure (BP) abnormalities such as orthostatic hypotension (OH), postprandial hypotension, and nocturnal hypertension are well documented [3-9]. Posterior reversible encephalopathy syndrome (PRES) is a clinico-radiological entity characterized by headache, altered mental status, seizures, and visual disturbances, along with neuroimaging findings of vasogenic edema predominantly in the parieto-occipital regions. The pathophysiology of PRES is not completely understood but is thought to involve disordered autoregulation of cerebral blood flow leading to hyperperfusion, endothelial dysfunction, and fluid extravasation into the brain parenchyma [10-16]. The association between PD, BP fluctuations, and PRES has not been extensively reported in the literature. This case report aims to highlight this potential relationship and discuss the clinical implications.

This case report was previously posted on the Research Square preprint server on December 9, 2025.

## Case presentation

**Figure 1:**
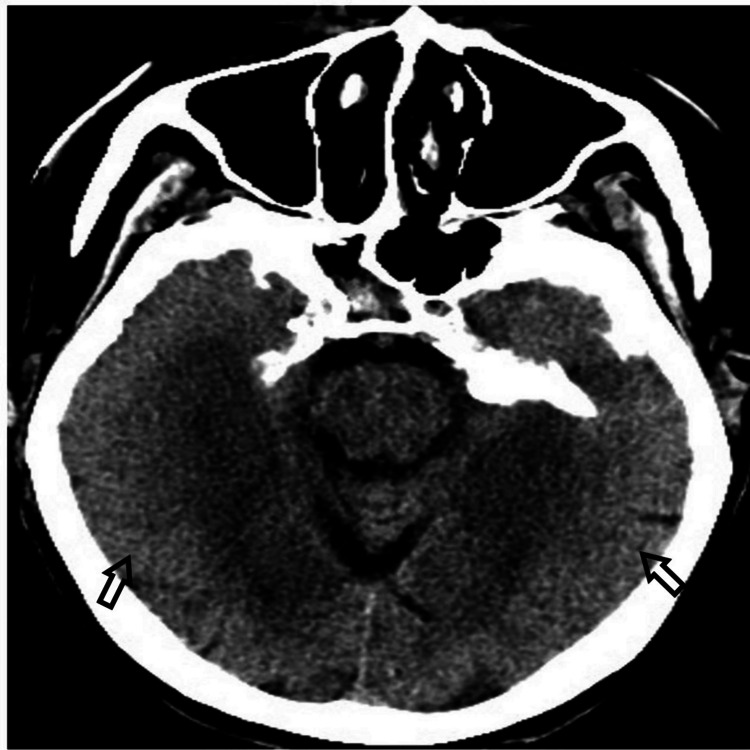
Bilateral symmetrical medial occipito-temporal lobes predominantly in the subcortical white matter with lesser extent cortical hypodensities.

**Figure 2:**
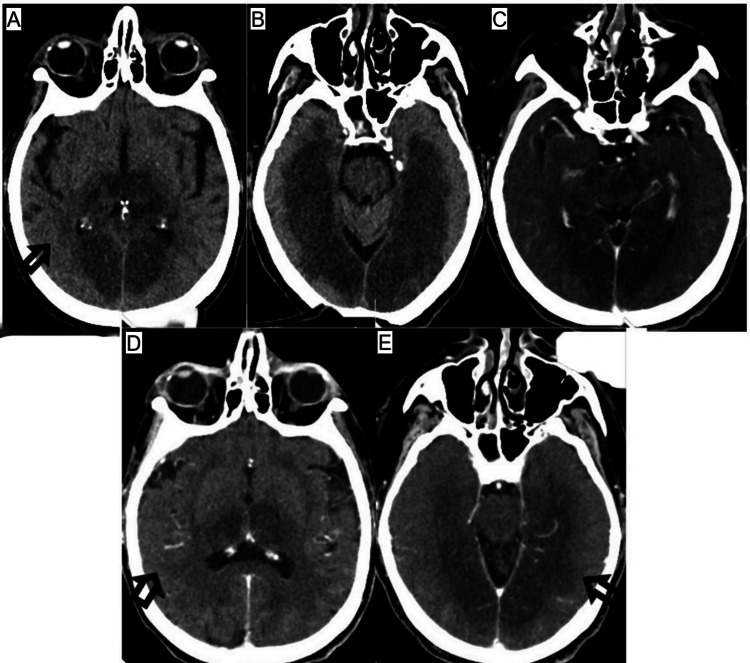
(A-E) Progression of the bilateral medial occipito-temporal lobe hypodensities with bilateral thalami involvement. Patent basilar and bilateral posterior cerebral arteries.

**Figure 3:**
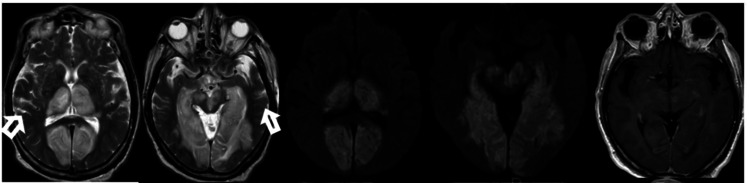
Bilateral medial occipito-temporal lobes, thalami, and midbrain T2 hyperintensities with diffusion restriction, foci of susceptibility effect, and patchy enhancement.

The case describes an 82-year-old woman with a 10-year history of PD and severe autonomic dysfunction who presented with an altered level of consciousness and a severe headache following a home hypertensive peak of 210/115 mmHg. At the time of presentation, the patient’s medication regimen for PD included levodopa/carbidopa 100/25 mg TID and pramipexole 0.5 mg TID. No recent dosage escalations or additions of sympathomimetics were reported. Although her BP was recorded at 119/79 mmHg upon emergency department admission, her neurological status was critical with a Glasgow Coma Scale (GCS) of 3 (E1, V1, M1), necessitating immediate intubation. While the initial GCS assessment following 24 hours of fentanyl sedation may have been confounded by residual drug effects, subsequent neuroimaging confirmed a diagnosis of PRES. Specifically, brain magnetic resonance imaging (MRI) revealed T2/fluid-attenuated inversion recovery (FLAIR) hyperintensities in the bilateral parieto-occipital regions and demonstrated focal diffusion restriction, suggesting a progression from vasogenic to cytotoxic edema, which indicates a more severe insult and a guarded prognosis (Figures 1-3).

Serial inpatient monitoring confirmed continued autonomic instability, with readings fluctuating between 190/110 and 105/65 mmHg. Despite a gradual improvement in her GCS to 7/15 (E2, V1, M4) over two weeks, the patient required a tracheostomy for prolonged airway management, illustrating that the profound BP dysregulation seen in advanced PD can trigger rare, life-threatening complications like PRES with cytotoxic conversion.

## Discussion

Autonomic dysfunction and BP abnormalities are well-recognized features of PD; however, their potential role in the development of PRES remains unclear and is rarely reported in the literature. To define, BP fluctuations include OH, supine hypertension, or loss of circadian BP regularity, and are symptoms of dysautonomia in PD [12]. Alternating OH and supine hypertension during the day, “reduced dipping,” and loss or return of the typical drop in BP during sleep, “reverse dipping,” are included when BP fluctuation is mentioned [10].

Many articles have studied the association between BP fluctuations and PD [1,6,12]. In addition, a study mentioned a decrease in mean systolic BP (SBP) in patients with PD [15,17]. While there are several pathophysiological theories linking the two, some have focused on autonomic dysfunction in PD [12]. His study noted that circadian rhythm disruption in patients with PD is a major contributor to BP variation. Others agreed that sleep disturbances are a significant factor that may affect PD patients’ BP at night [1]. The patient’s 10-year history of PD suggests a likely presence of autonomic dysfunction. While direct measurements of circadian BP patterns or formal sleep studies were not available for this patient, it is worth noting that circadian rhythm disruption is common in advanced PD and can contribute to BP variability. Therefore, while hypothetical in this instance, such chronobiological factors may represent a potential underlying risk factor for the BP fluctuations that precipitate PRES. At this point, we carefully considered a diagnosis of multiple system atrophy (MSA). However, the patient lacked several key signs for MSA, such as early severe gait instability, disproportionate antecollis, or the “hot cross bun” sign on brain MRI. Furthermore, the patient’s sustained response to levodopa over the past years strongly supports a diagnosis of idiopathic PD over MSA.

In line with previously reported cases (Table 1), our patient’s long-standing PD with marked BP fluctuation resembles the clinical patterns described in PD-associated PRES [11]. Understanding how BP fluctuations contribute to PRES development is crucial for diagnosis and management. According to Hinduja, fluctuations in BP are a common cause of PRES [5]. This is due to the distribution of cerebral autoregulation. An abrupt surge in BP overwhelms the compensatory response of cerebral vessels, leading to hyperperfusion, disruption of the blood-brain barrier, and vasogenic edema. The posterior circulation is especially vulnerable due to the reduced sympathetic innervation. Moreover, BP fluctuations are associated with an increased risk of PRES, rather than mere BP elevation. This pathophysiological cascade is characteristic of PRES and aligns with the patient’s acute presentation with headache, reduced level of consciousness, and low GCS. In turn, the interaction between PD-related dysautonomia and chronic BP variability provides a reasonable explanation for the development of PRES in our case of an 82-year-old patient in the absence of usual triggers like vasopressors [5].

**Table 1:** Comparison of published PRES case reports and investigations of PD-related autonomic dysfunction [1,2,5-7,10-13]. PRES: posterior reversible encephalopathy syndrome; PD: Parkinson's disease; BP: blood pressure

Author(s), year	Type (case/study/review)	Patient(s)/sample size, age & sex	Key details (BP variability, PD, PRES relationship) and outcome	Reference number
Alves et al., 2023	Case-control study	-	Suggests that PD patients may present a higher BP variability	[1]
Fugate and Barrett, 2014	Review	-	Suggested that PRES can be caused by BP fluctuation, regardless of the underlying cause	[2]
Hinduja, 2020	Review of PRES mechanisms	-	Established rapid BP rise and impaired autoregulation as the core mechanism of PRES; highlighted posterior circulation vulnerability due to less sympathetic innervation	[5]
Kanegusuku et al., 2017	Case-control	21 patients with PD	Patients with PD present a blunted nocturnal BP fall and similar ambulatory blood pressure variability (ABPV), assessed by standard deviation	[6]
Shen et al., 2022	Cross-sectional	32 patients with PD	Noted that reverse dipping (a pattern of BP variability) was more common in PD patients in this study, especially in the advanced PD patients	[7]
Milazzo et al., 2018	Cross-sectional study	-	Studied the association between abnormal circadian BP profile and autonomic dysautonomia in PD	[10]
Morozumi et al., 2016	Case report	77-year-old man with 10-year PD	PRES attributed to extreme BP fluctuation: orthostatic hypotension + supine hypertension; suggested PD-related autonomic failure as trigger. The only case with a similar background and presentation to our case	[11]
Pierzchlińska et al., 2021	Review	-	Studied the effect of circadian rhythm on BP variability in patients with PD	[12]
Riley and Espay, 2018	Case report	86-year-old man with PD	BP variability in PD contributes to end-organ damage, including cognition and possibly dysautonomia	[13]
Current case, 2025	Case report	82-year-old woman with PD	Presented with autonomic dysfunction. The authors suggest that untreated autonomic changes in PD may lead to PRES	New

Despite some reports’ efforts to highlight the link between BP variability and cerebrovascular complications in PD, or the association with age, only a few focus on the relationship between BP variability, PD, and PRES (Table 1). Our case highlights the possibility of PRES being caused by BP variability (not confined to orthostatic) in a patient with long-standing PD.

## Conclusions

This case demonstrates that long-standing PD, characterized in our patient by severe motor fluctuations and documented OH, can lead to catastrophic neurovascular outcomes. Despite a stable management plan including levodopa/carbidopa and dopamine agonists, the patient developed PRES with cytotoxic conversion, likely driven by profound autonomic-mediated BP dysregulation. Clinicians must maintain a high index of suspicion for PRES in PD patients presenting with acute neurological decline, as early recognition is vital to prevent irreversible damage. This case highlights the need for aggressive monitoring of BP variability, rather than just mean values, in the advanced stages of the disease.
